# When do cover crops reduce nitrate leaching? A global meta‐analysis

**DOI:** 10.1111/gcb.16269

**Published:** 2022-06-08

**Authors:** Amin Nouri, Scott Lukas, Shikha Singh, Surendra Singh, Stephen Machado

**Affiliations:** ^1^ Hermiston Agricultural Research and Extension Center Oregon State University Hermiston Oregon USA; ^2^ Columbia Basin Agricultural Research Center Oregon State University Adams Oregon USA

**Keywords:** brassica, cover crop, grass, legume, nitrate leaching, no‐tillage, soil order, soil texture

## Abstract

The global increases in the surface and groundwater nitrate (NO_3_^−^) concentrations due to synthetic fertilizer input have emerged as major sustainability threats to terrestrial and aquatic ecosystems. Cover crops can reportedly reduce nitrate leaching from croplands. However, the underlying mechanisms and the effectiveness of cover crops in reducing nitrate leaching across species, soil types, agronomic management, and climates remain elusive. We conducted a global meta‐analysis to evaluate the effects of cover crops on nitrate leaching and water drainage. A random‐effects analysis was established to investigate seven moderating variables in 41 articles. Results showed that globally, cover crops reduced nitrate leaching by 69% compared with fallow while demonstrating no effect on water drainage. Overall, cover crops from Brassicaceae and Poaceae families showed the greatest effect with 75% and 52% reduction in nitrate leaching, respectively. Cover cropping on Ultisols, Histosols, and Inceptisols resulted in the greatest reduction in nitrate leaching (77%, 78%, and 77%, respectively). Greater efficacy of cover crops at reducing nitrate leaching was evident with increasing soil sand content. In general, cover crops appeared to perform better to reduce nitrate leaching in vegetable systems compared to field crops. Cover cropping on conventional tillage resulted in a 63% reduction in nitrate leaching compared with no‐tillage (50%) and reduced tillage (38%) systems. The impact of cover crops on water drainage was nonsignificant which implies that nitrate leaching control by cover crops is unlikely exerted through reducing water drainage. This study brings further insight into the intrinsic factors affecting cover crop efficacy and management practices that enhance cover crop potential in reducing nitrate leaching from agricultural systems.

## Graphical abstract

Contamination of surface and groundwater by synthetic nitrogen fertilizers is an environmental challenge in many regions of the world. The current meta‐analysis reveals that cover cropping is an effective strategy to reduce nitrate leaching to deeper soil profiles and discusses the climatic, terrestrial, and managerial conditions that influence the efficacy of cover crops in reducing nitrate leaching.

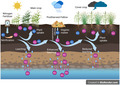

## 1 INTRODUCTION

Nitrate (NO_3_^−^–N) from synthetic and livestock‐derived fertilizers, at rates beyond efficient nitrogen use is a major source of freshwater contamination (Cameron et al., 2013; Fowler et al., 2013; Sacchi et al., 2013). Nitrate anions are repelled by soil cation exchange complex, thus mobilized with irrigation or precipitation water. Nitrate percolating below the root zone may not considerably subject to degradation (Chen et al., 2018; Hillel, 1998; Mergel et al., 2001; Peterson et al., 2013), uptake by plants, or atmospheric diffusion, thus moves freely downward along with the moisture front. For example, in potato production systems of the northern‐central USA, nitrate leaching accounted for 66% of the N fertilizer application (Stites & Kraft, 2001). Nitrate accumulation in groundwater may render the water unsafe for drinking, while its migration to surface waters may impair aquatic ecosystems.

The leaching process in farmlands is primarily controlled by soil nitrate concentration and water/solute flux. Monitoring water balance in the root zone by adopting efficient irrigation management can reduce nitrate leaching. However, increasing water use efficiency in many regions coincides with the expansion of irrigated areas (Lall et al., 2020). Additionally, in many agricultural systems, periodic overirrigation is inevitable to avoid the accumulation of excess salts in the root zone (Rezapour, Asadzadeh, et al., 2021). Global warming and subsequent increase in summer droughts are expected to amplify soil's excess salt accumulations (Rezapour, Nouri, et al., 2021). In recent decades, diminishing freshwater resources, particularly in arid and semi‐arid regions has led to the expansion of reuse water for irrigation. The nitrogenous compounds in alternative irrigation water resources may increase nitrate leaching. Likewise, the projected increase in high intensity—low‐frequency offseason precipitation events, also known as “hot moments” can further aggravate nitrate transport to water bodies by accelerating solute percolation and runoff generation (Reichstein et al., 2003). Thus, ensuring the optimum nitrogen input is important to minimize nitrate leaching under increasing climate perturbations (Nouri et al., 2021).

Field crops uptake around 50% of the N applied as fertilizer (Bundy & Andraski, 2005), and the residual N after crop uptake and gaseous losses is prone to leaching (Quemada et al., 2013; Russo et al., 2017; Thapa & Chatterjee, 2017; Zhao et al., 2016). Fall precipitation events following the cash crop harvest potentially increase nitrate leaching (Russo et al., 2017). There is no crop to uptake the leftover N during fallow periods, and biological denitrification is curtailed by decreasing soil temperature (except for tropics). Thus, transport of surplus N after cash crop harvest may continue over the fallow period if rainfall persists.

Replacing fallow with cover crops can minimize the nitrate leaching from croplands (Gabriel & Quemada, 2011; Meisinger & Ricigliano, 2017; Singh, Schoonover, et al., 2018). Cover crops can reduce nitrogen losses by taking up both residual soil N and water (Li et al., 2021). Utilization of soil water by the cover crops reduces the water available to leach nitrate, while nitrogen utilized by cover crops eventually becomes available to the subsequent crop after the termination of the cover crop. Many studies report that winter cover crops including nonlegumes such as grasses and broadleaf species could reduce nitrate leaching by 35%–70% depending on soil, climate, and management strategies (Quemada et al., 2013) while Kladivko et al. (2014) estimated the reduction in leaching losses ranged from 13% to 94% with cover crops. Additionally, cover crops have been shown to reduce the raindrop impact and subsequent soil surface sealing that may intensify nitrate transport by runoff. In a critical global review, Abdalla et al. (2019) concluded that adopting cover crops, besides reducing nitrate leaching, increases soil carbon sequestration with no significant contribution to N_2_O emissions.

A range of natural and engineered processes have been developed for groundwater nitrate remediation. In general, these methods are based on groundwater pump and treat techniques such as bacterial denitrification, adsorbents, membranes (i.e., osmotic and bioreactor), and electro‐dialysis (Huno et al., 2018). In contrast to cover cropping, these methods target the nitrate that has already migrated from the soil profile into the groundwater. Most of these methods are cost‐intensive and usually generate by‐products that require secondary treatment (Huno et al., 2018).

In addition to cover crops, no‐tillage (NT) has been shown to reduce nitrate leaching (Baker, 2001; Daryanto et al., 2017; Spiess et al., 2020). Reduced nitrate leaching under NT is attributed to increased denitrification losses through the increased water retention capacity (Senigagliesi & Ferrari, 1993). However, the effect of tillage on nitrate leaching is confounding as the NT management duration is a key determinant (Nouri et al., 2020), and the response may vary broadly across soil types. Thus, some studies have reported that NT reduces nitrate leaching (Randall & Iragavarapu, 1995; Zhu et al., 2003), while some reported little to no effect of NT on nitrate leaching (Al‐Kaisi & Licht, 2004; Singh, Williard, et al., 2018; Spiess et al., 2020). Nonetheless, there is a lack of information regarding the individual and synergistic effects of cover crops and NT on water drainage and nitrate leaching.

The efficacy of cover crops in reducing nitrate leaching may vary across crop species and genera. For instance, cereal rye (*Secale cereale* L.) is a preferred winter cover crop for residual scavenging soil N after harvesting field crops, thereby reducing N leaching. Teixeira et al. (2016) reported a greater efficacy of nonlegume versus legume cover crops in reducing nitrate leaching. The essential driver of cover crop efficacy is the interaction between agronomic practices and cover crop species with climatic and terrestrial factors. Soil texture, for instance, is a key determinant of contact time between nitrate in solution and the biotic and abiotic sinks that control the availability of inorganic N for cover crop uptake (Lin et al., 2005). However, evaluating the nitrate leaching potential only based on soil texture may neglect the effect of soil structure on the biogeochemical processes that control N dynamics. Thus, understanding the effect of cover crops on nitrate leaching across soil orders can provide additional information regarding the possibility of pedogenic (e.g., redox reactions, restrictive horizons, organic matter, preferential flow, and eluvial processes) influence on plant N uptake process.

A global meta‐analysis on synchronized water drainage and nitrate leaching can improve our understanding of the mechanisms (e.g., biogeochemical or hydrologic) through which cover crops regulate nitrate leaching across climates and soils. In a meta‐analysis, Tonitto et al. (2006) compared the crop yield and nitrate leaching between winter fallow and legume or nonlegume cover crops. They reported 70% and 40% decrease in nitrate leaching when winter fallow was replaced with nonlegume and legume cover crops, respectively. In an updated meta‐analysis, Thapa et al. (2018) found that nonlegume cover crops reduce nitrate leaching by 56%. They found that the legume–nonlegume mixtures were as effective as non‐legumes but were significantly more effective than legume cover crop species. Nonlegume cover crop species can substantially reduce nitrate leaching below the root zone compared to legumes. However, there is little information on the efficacy of different nonlegume cover crop species which have been frequently documented with a greater potential to reduce nitrate leaching. Additionally, due to the lack of coupled nitrate leaching and water drainage data reported in two other meta‐analyses, the underlying mechanism through which cover crops control nitrate leaching (i.e., flow regulation vs. nitrate uptake) is ambiguous. Furthermore, the additional data included in the current meta‐analysis enabled the investigation of cover cropping effect on nitrate leaching across a wide range of plant families and genera, soil orders and main crops which is lacking in the previous meta‐analyses. To this end, we conducted a global meta‐analysis including the most updated body of research on the effect of cover crops on both nitrate leaching and water drainage. Our main objectives were: (1) to assess the effect of cover crop types on nitrate leaching on across different soil textures, soil orders, tillage practices, and climates; and (2) to investigate cover crop effects on coupled nitrate leaching and water drainage.

## 2 MATERIALS AND METHODS

### 2.1 Data collection

**FIGURE 1:**
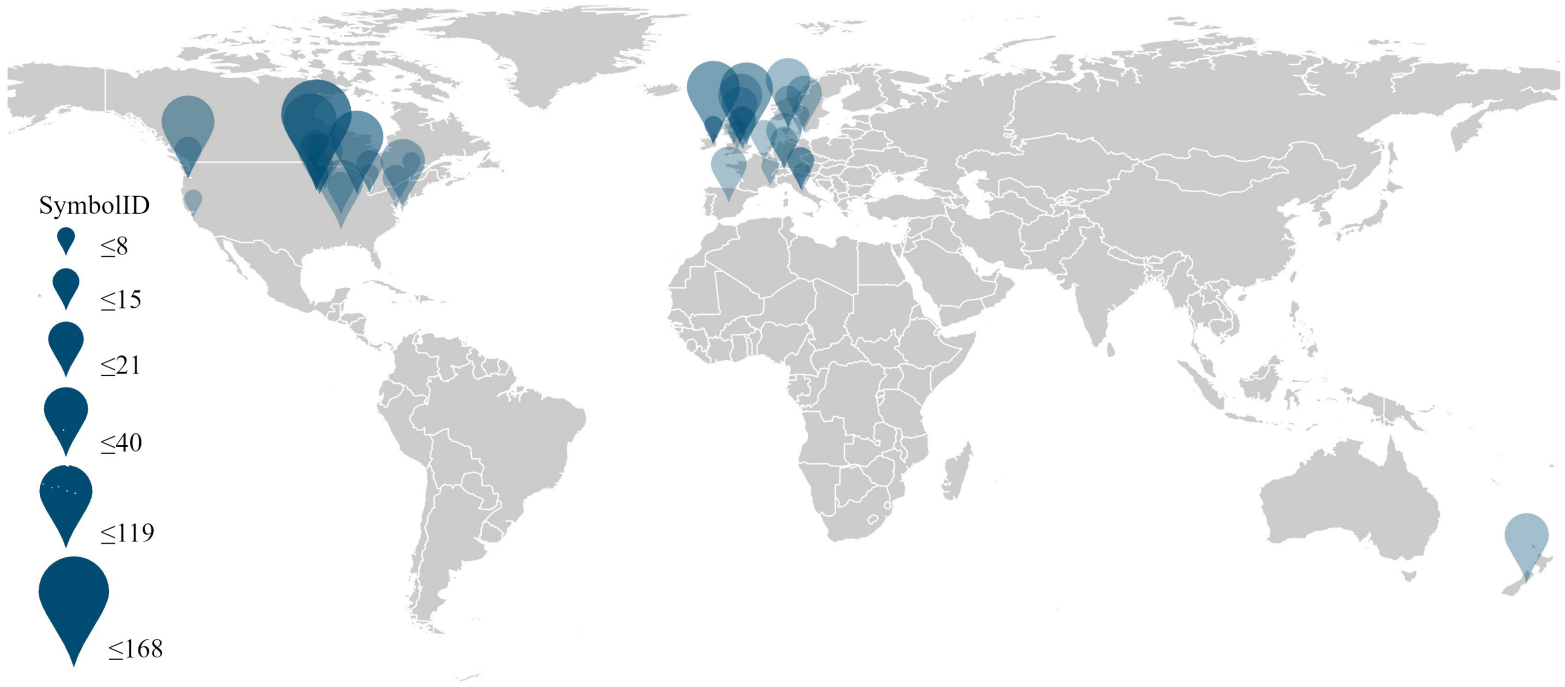
Graduated symbol map representing the geographic distribution of studies and number of observations (*n*).

Data collection was conducted in 2021, on the Web of Science Core Collection using the ISI Web of Science® search engine. The search term “cover crop” resulted in 4624 individual publications. A search for “nitrate” AND “leaching” within this record resulted in 231 publications including articles, review papers, conference proceedings, early access, and book chapters. To ensure the full inclusion of relevant papers, the mentioned keywords were formulated as follows: (“cover crop*” OR “biocover*”) AND (“nitr* leach*” OR “nitrogen leach*” OR “nitr* drain*” OR “nitrogen drain*”). The resulting publications were narrowed down to 41 publications by excluding the articles that did not meet our criteria. The excluded articles were those that reported cover crop means without reporting means for NCC treatment, articles that did not report nitrate loading through analyzing leachate (soil N was reported instead), and indoor experiments. Likewise, duplicate papers and those lacking experimental data (review or book chapter) and complete information on soil texture, study location, and experiment design were excluded. Also, proceedings (only the abstract was available) and papers published before 1931 or articles written in languages other than English were not included. The NCC treatment was compared to each cover cropping system separately for unbalanced experimental designs or where multiple cover cropping systems were conducted. For studies reporting the overall mean without mentioning the number of replicates, *n* = 1 was considered, unless the study error or least significant difference was provided. WebPlotDigitizer® (Rohatgi, 2014) was used to extract data if results were given as graphics. Data from tables were extracted using Tabula, version 1.2.1 (Aristarán et al., 2018). The global distribution of studies and associated weights are provided in Figure 1.

Multiple observations of nitrate leaching (multiple years, seasons, varieties, etc.) from a singular publication were treated as independent reports. This approach has the advantage of increasing the statistical power of meta‐analysis (Ferraretto & Shaver, 2015; Toler et al., 2019). Additionally, observations across varying annual, seasonal, and varietal factors comprise different environmental conditions and genetic characteristics. Therefore, total number of 1119 independent observations were derived from 41 articles that are provided in the Appendix S1.

### 2.2 Effect size and moderators

The meta‐analysis was conducted for two response variables: (i) nitrate leaching and (ii) water drainage. Reports on total nitrogen losses comprised both the nitrate concentration of the soil solution and the amount of drained water. Hence, the impact of cover crops on water drainage provides valuable insight into the mechanism through which cover crops regulate nitrate losses from agricultural systems. The overall effect size (ES) of each variable was calculated as the natural logarithm of the response ratio of the cover crop to the NCC control:

(1) $$\mathrm{ES}=\ln\left(R\right)=\ln\left(\frac{{\bar{X}}_{\mathrm{CC}}}{{\bar{X}}_{\mathrm{NCC}}}\right)$$

where ES is the effect size, $\bar{X}$_CC_ is the cover crop mean, and $\bar{X}$_NCC_ is the mean of the NCC control (Rosenberg et al., 1997). The use of the response ratio in biological studies provides a standardized definition of treatment effect. The natural logarithm on the other hand maintains a symmetry in the analysis by balancing the negative and positive treatment effects across response ratios (Borenstein et al., 2007). Thus, the magnitude of response ratio values (> zero) indicates the extent of variables' response to cover crop. To facilitate the result interpretation, the natural logs of ESs were back transformed to raw ratios and provided as %Change within the forest plots.

To understand the environmental and physiological factors that may influence the response of nitrate leaching and water drainage to cover crops, we collected information on a suite of moderators (Table 1). Each moderator comprised at least two levels, each collected from at least three articles. We aimed to identify how differences in cover crop types, soil characteristics, and main crop may affect the effectiveness of cover crops in regulating nitrate leaching and water drainage. Among moderator variables, soil classification for non‐US studies was adapted to USDA Soil Taxonomic System (Science & Administration, 1975). The tillage effect was assessed by comparing the effect sizes (CC/NCC) of nitrate leaching and water drainage under conventional tillage (CT), reduced tillage (RT), and no‐tillage (NT) practices. In addition to the categorical moderators, the effect of numerical variables including rainfall and air temperature was assessed by meta‐regression analysis. Meta‐regression is based on a similar approach as in primary regression analysis except that covariates are studies rather than subjects and the dependent variable is the effect size rather than subject scores. We aimed to identify if the covariates that significantly affect the effect size. Considering the likelihood of true effect variability across studies, meta‐regression analysis was conducted using a random‐effects model (restricted maximum likelihood method).

**TABLE 1:** Moderator analysis of cover crop effects on nitrate leaching

Moderator	*n*	*Q*‐value	df (*Q*)	*p*_hetero_	*I* ^2^ (%)
Cover crop family					
Poaceae	348	433	347	.001	19.8
Brassicaceae	132	943	131	.000	86.1
Leguminosae	46	71	45	.009	36.3
Asteraceae	10	2	9	.986	0.0
Multigenera	55	54	54	.483	0.0
Total within		1502	586	.000	
Total between		118	4	.000	
Overall	591	1621	590	.000	63.6
Cover crop genus					
Avena	19	105	18	.000	82.8
Hordeum	21	23	20	.292	12.8
Lolium	5	1	4	.971	0.0
Poa	6	3	5	.763	0.0
Secale	277	277	276	.470	0.4
Triticum	20	4	19	1.000	0.0
Brassica	84	862	83	.000	90.4
Camelina	12	6	11	.865	0.0
Raphanus	24	16	23	.859	0.0
Thlaspi	12	6	11	.895	0.0
Trifolium	7	0	6	.999	0.0
Vicia	39	70	38	.001	45.7
Total within		1428	577	.000	
Total between		193	13	.000	
Overall	591	1621	590	.000	63.6
Soil order					
Ultisols	9	6	8	.684	0.0
Mollisols	273	98	272	1.000	0.0
Histosols	44	88	43	.000	51.1
Alfisols	9	27	8	.001	70.4
Inceptisols	95	968	94	.000	90.3
Entisols	46	25	45	.994	0.0
Total within		1212	470	.000	
Total between		226	5	.000	
Overall	476	1438	475	.000	67.0
Soil texture					
Clay	6	0	5	.999	0.0
Silty clay loam	48	23	47	.999	0.0
Clay loam	146	170	145	.080	14.5
Silt loam	82	178	81	.000	54.6
Loam	102	131	101	.025	22.6
Sandy loam	146	976	145	.000	85.1
Loamy sand	46	25	45	.994	0.0
Sandy	12	8	11	.692	0.0
Total within		1511	582	.000	
Total between		110	8	.000	
Overall	588	1621	590	.000	63.6
Main crop					
Tomato	10	3	9	.975	0.0
Potato	7	4	6	.636	0.0
Broccoli	12	1	11	1.000	0.0
Sugar beet	7	3	6	.781	0.0
Sunflower	9	7	8	.582	0.0
Soybean	80	23	79	1.000	0.0
Corn	139	170	138	.033	18.8
Wheat	23	13	21	.901	0.0
Barley	114	1014	113	.000	88.9
Other beans	6	0	5	.997	0.0
Crop rotation	37	44	36	.180	17.4
Total within		1285	440	.000	
Total between		123	14	.000	
Overall	455	1408	454	.000	67.8

Abbreviations: df, within‐moderator degree of freedom; *I* ^2^, the ratio of true variation to total variation; *n*, number of studies; *p*_hetero_, probability that total heterogeneity is the result of sampling error; *Q*, weighted sum of squared differences between the observed effects and the weighted average effect.

### 2.3 Meta‐analysis

The meta‐analysis was conducted based on the methodology of Borenstein et al. (2007) and followed the recommendations by Koricheva and Gurevitch (2014). Considering the likelihood of true effect variability across studies, the random‐effects model was selected for this meta‐analysis. The analyses were conducted in Comprehensive Meta‐Analysis (CMA) software (Borenstein et al., 2013). As a common shortage, a considerable number of studies did not report the study variance (e.g., standard error or standard deviation) or sufficient information was not provided to obtain the internal dispersion from the Least Significance Difference (Toler et al., 2019; Veresoglou et al., 2012). Therefore, individual studies were weighted using nonparametric variance:

(2) $$V_{\ln\left(R\right)}=\frac{\left(n_{\mathrm{CC}}+n_{\mathrm{NCC}}\right)}{\left(n_{\mathrm{CC}}\times n_{\mathrm{NCC}}\right)}$$

where *V*_ln(*R*)_ is the variance of the natural log of the response ratio *R*, and *n*_CC_ and *n*_NCC_ are the sample sizes of the cover crop and NCC treatments (Rosenberg, 2000). We used *Q* statistics (weighted squared deviation) to assess the heterogeneity, which was quantified using *I* ^2^, the ratio of true heterogeneity to total observed variation:

(3) $$I^{2}=\frac{\left(Q_{\text{total}}-\mathrm{df}\right)}{Q_{\text{total}}}\times 100$$

where *Q*_total_ is total variation, df is the degree of freedom (within‐study variation), and *Q*_total_–df is true heterogeneity or between‐study variation. *I* ^2^ values range between 0% and 100%, 0% indicating no true heterogeneity and greater values representing a larger fraction of the observed variation due to between‐study variation in the dataset. The null hypothesis for heterogeneity is that the studies share a common effect size. The *p*‐value <.1 due to the low‐power nature of the *Q*‐test implies the invalidity of the homogeneity assumption (Toler et al., 2019). The term “significant” throughout the text indicates a *p*‐value that is less than the confidence interval.

## 3 RESULTS

### 3.1 Heterogeneity and publication bias

**FIGURE 2:**
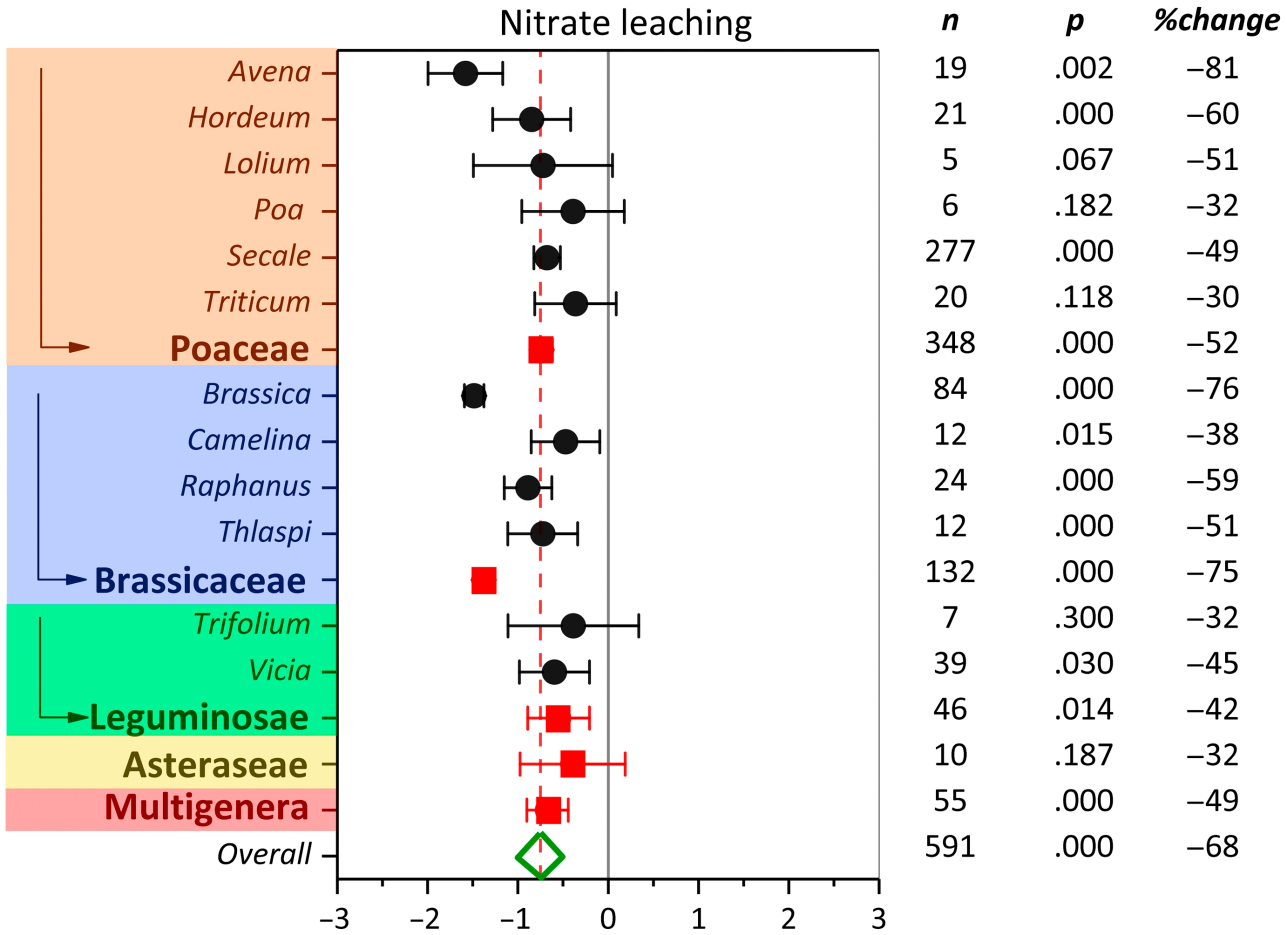
Weighted summary effect sizes (ln(*R*)) and 95% confidence intervals for cover crop effect on nitrate leaching as influenced by cover crop family and suborder plant genus. %Change refers to the effect size differences from the overall mean. *n* is the number of studies. *p*‐value <.05 indicates that the summary effect size is different than zero.

We examined the effect of cover crop (CC/NCC response ratio) on nitrate leaching by analyzing a global dataset extracted from 41 studies. The cover crop was represented by five plant families and 12 plant genera (Figure 2). Table 1 provides the *Q* statistics, a measure of weighted square deviations at moderator, and overall levels for nitrate leaching effect size. The overall heterogeneity was significant (*p*_hetero_ < .10) for all five moderators, even though the *p*_hetero_ varied across moderators' subgroup levels. The overall *I* ^2^ ranged between 63.6% and 67.8% representing the percent of variability which is due to heterogeneity rather than chance. The metrics used to assess the publication bias were within the acceptable ranges. The funnel plot illustrated an expected variability (standard error) of small and large studies around the common effect size. According to Begg and Mazumdar rank correlation test, the effect size had an absolute Kendall *τ* value (~−0.01) <0.20 threshold (two‐tailed *p* = .71), suggesting no evidence of publication bias or no tendency for effect size to increase as study size decreased. Heterogeneity statistics for water drainage under the influence of five moderators are shown in Table 2. The *Q*‐statistics shows no significant overall heterogeneity at five moderator levels for water drainage (*p*_hetero_ <.10). The overall *I* ^2^ for all moderators were 0.00% indicating the lack of true heterogeneity. Among moderator subgroups, *Hordeum* genus (*p*_hetero_ = .051, *I* ^2^ = 38.3), silty clay loam soil texture (*p*_hetero_ = .018, *I* ^2^ = 49.8), and corn (*Z. Mays*) main crop (*p*_hetero_ = .083, *I* ^2^ = 18.5) demonstrated significant heterogeneity (Table 2). Begg and Mazumdar rank correlation test (*τ* = 0.03, two‐tailed *p* = .46) did not show evidence of publication bias.

**TABLE 2:** Moderator analysis of cover crop effect on water drainage

Moderator	*n*	*Q*‐value	df (*Q*)	*p*_hetero_	*I* ^2^
Cover crop family					
Poaceae	203	191	202	.702	0.0
Brassicaceae	16	0	15	1.000	0.0
Leguminosae	11	1	10	1.000	0.0
Multigenera	10	3	9	.976	0.0
Total within		195	236	.977	
Total between		1	3	.886	
Overall	240	195	239	.982	0.0
Cover crop genus					
Avena	5	0	4	1.000	0.0
Hordeum	18	28	17	.051	38.3
Poa	4	0	3	.995	0.0
Secale	154	155	153	.449	1.0
Triticum	21	2	20	1.000	0.0
Brassica	6	0	5	1.000	0.0
Raphanus	10	0	9	1.000	0.0
Vicia	10	1	9	1.000	0.0
Multigenera	10	3	9	.976	0.0
Total within		188	229	.979	
Total between		8	8	.470	
Overall	238	195	237	.978	0.0
Soil order					
Ultisols	9	0	8	1.000	0.0
Mollisols	152	89	151	1.000	0.0
Alfisols	14	3	13	.999	0.0
Inceptisols	24	30	23	.164	22.1
Entisols	12	1	11	1.000	0.0
Total within		122	206	1.000	
Total between		14	4	.009	
Overall	211	136	210	1.000	0.0
Soil texture					
Silty clay loam	14	26	13	.018	49.8
Clay loam	119	79	118	.998	0.0
Silt loam	34	2	33	1.000	0.0
Loam	10	1	9	1.000	0.0
Sandy loam	48	41	47	.710	0.0
Loamy sand	12	1	11	1.000	0.0
Total within		151	233	1.000	
Total between		45	6	.000	
Overall	237	195	239	.982	0.0
Main crop					
Tomato	10	3	9	.964	0.0
Soybean	63	5	62	1.000	0.0
Crop rotation	6	0	5	1.000	0.0
Corn	80	97	79	.083	18.5
Barley	22	1	21	1.000	0.0
Others	8	1	7	.997	0.0
Total within		107	183	1.000	
Total between		4	5	.538	
Overall	189	111	188	1.000	0.0

Abbreviations: df, within‐moderator degree of freedom; *I* ^2^, the ratio of true variation to total variation; *n*: number of studies; *p*_hetero_, probability that total heterogeneity is the result of sampling error. *Q*, weighted sum of squared differences between the observed effects and the weighted average effect.

### 3.2 Cover crop effect on nitrate leaching

Overall summary effect size across all families and genera demonstrated the significant effect of cover crops on reducing nitrate leaching. Moderator analysis indicated that among cover crop families the most information was available for Poaceae (348 observations). Brassicaceae, the second most studied cover crop family (132 observations) had the major role in the significant overall summary effect. Among Brassicaceae, *Brassica* (i.e., mustard, stubble turnip, white mustard, forage rape) and *Raphanus* genera (i.e., Graza radish, and oilseed radish) reduced nitrate leaching by 76% and 59% compared with NCC control, respectively. Individually, *Brassica* and *Raphanus* genera exhibited significantly greater effect size than pooled effect size on reducing nitrate leaching (Figure 2). Compared with NCC, among Poaceae genera, *Avena* (i.e., oat), *Hordeum* (i.e., barley), and *Secale* (i.e., cereal rye) reduced nitrate leaching by 81%, 60%, and 49%, respectively, even though their effects were not significantly greater than the summary effect size. Likewise, the overall effect of Leguminosae genera on reducing nitrate leaching was significant, which was primarily due to the substantial contribution of *Vicia* (i.e., vetch) compared to *Trifolium* (i.e., white clover, Kura clover, subterranean clover) genera. Overall, the Multigenera (mixture) cover crops were as effective as Poaceae genera in reducing nitrate leaching. Asteraceae (i.e., sunflower) was the only cover crop family with no significant impact on reducing nitrate leaching (Figure 2).

**FIGURE 3:**
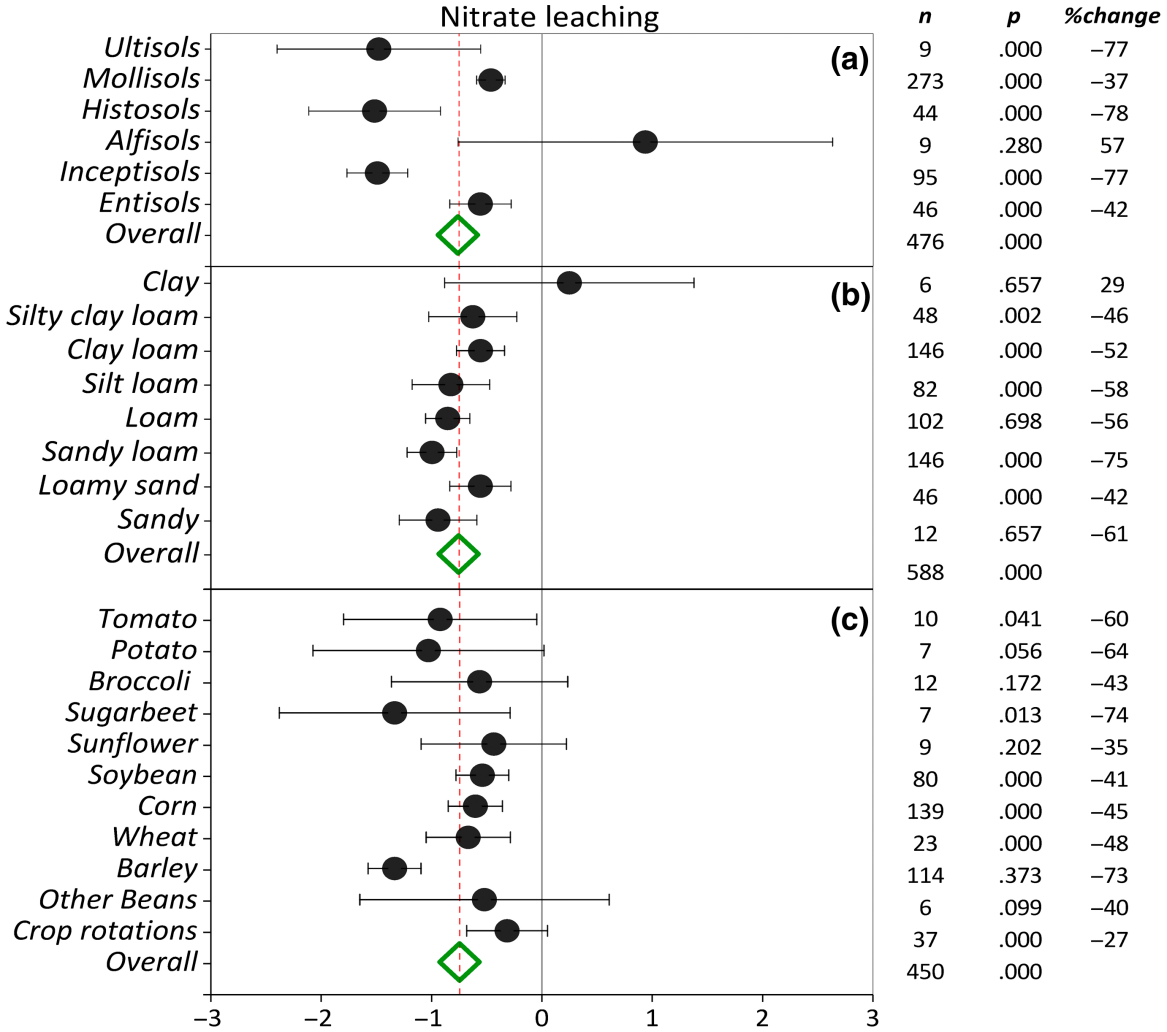
Weighted summary effect sizes (ln(*R*)) and 95% confidence intervals for cover crop effect on nitrate leaching as influenced by (a) soil order, (b) soil texture, and (c) main crop. %Change refers to the effect size differences from the overall mean. *n* is the number of studies. *p*‐value <.05 indicates that the summary effect size is different than zero.

**FIGURE 4:**
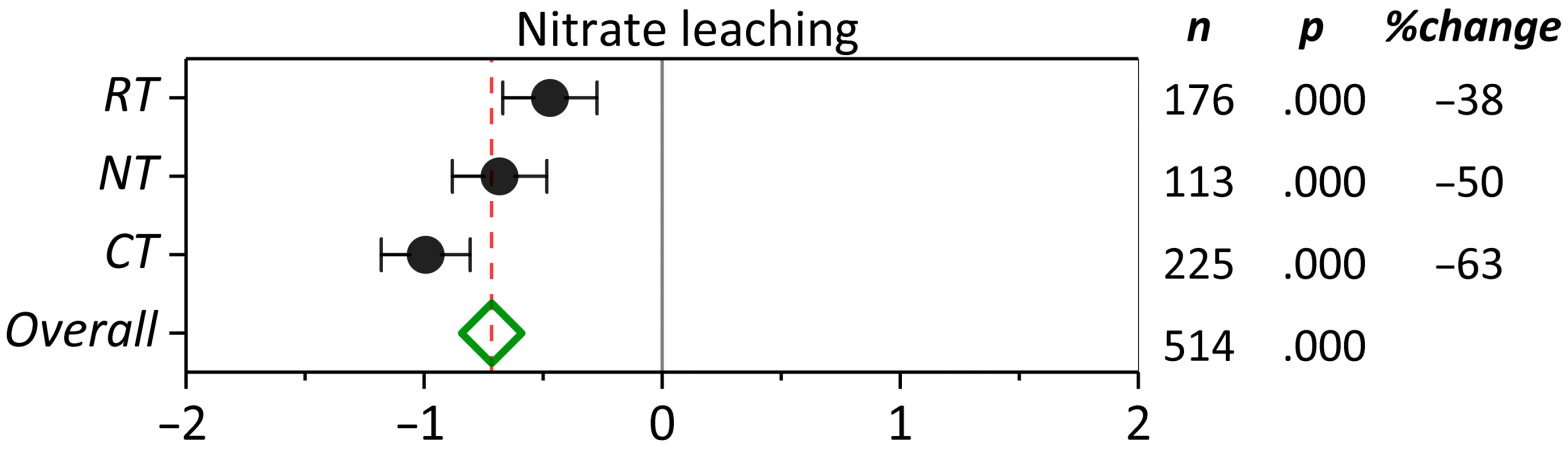
Weighted summary effect sizes (ln(*R*)) and 95% confidence intervals for cover crop effect on nitrate leaching as influenced by tillage management. %Change refers to the effect size differences from the overall mean. *n* is the number of studies. *p*‐value <.05 indicates that the summary effect size is different than zero.

Soil orders included in the literature have been sorted based on the descending level of weathering in the *y*‐axis of Figure 3a. Among six soil orders, cover cropping in Alfisols suggested a possible increase in nitrate leaching (*p* > .05) (Figure 3a). Compared with NCC, cover cropping in Ultisols, Mollisols, Histosols, Inceptisols, and Entisols resulted in 77%, 37%, 78%, 77%, and 42% reduction in nitrate leaching, respectively (*p* < .05). The greatest effect sizes were observed in Ultisols, Histosols, and Inceptisols, while the highest confidence was represented by narrower confidence intervals in Mollisols, Inceptisols, and Entisols. Among soil textural classes, cover cropping in clayey soils suggested a possible increase in nitrate leaching (*p* > .05) (Figure 3b). However, cover cropping reduced nitrate leaching significantly in all other textural classes. Among textural classes, cover cropping on sandy loam (75%) and sandy (61%) soils exhibited the greatest effect on the reduction of nitrate leaching compared with NCC. The soil textural classes included in the meta‐analysis have been sorted by decreasing soil clay content in Figure 3. A general trend of increasing cover crop efficacy in reducing nitrate leaching with increasing soil sand content was evident across soil textural classes (Figure 3b). In terms of the main crop effect, we observed relatively wider confidence limits compared to other moderators (Figure 3c). In general, horticultural crops, specifically tomato (*Solanum lycopersicum*), potato (*Solanum tuberosum*), and sugar beet (*Beta vulgaris*), demonstrated greater responses (*p* ≤ .05) than other main crops to the reduction in nitrate leaching by cover cropping. Among all main crops, cover crop adoption in barley systems resulted in the greatest reduction in nitrate leaching. Despite a positive response across all field and horticultural crops, wide confidence limits of sunflower and broccoli rendered their effects on nitrate leaching statistically nonsignificant. Likewise, crop rotations exhibited a smaller effect size and nonsignificant impact on reducing nitrate leaching compared with all monocropping systems. Cover cropping across tillage intensities significantly reduced nitrate leaching (Figure 4). Adoption of cover cropping in conventionally tilled systems resulted in a 63% decrease in nitrate leaching compared with 50% and 38% of reductions under NT and RT systems, respectively.

### 3.3 Cover crop effect on water drainage

**FIGURE 5:**
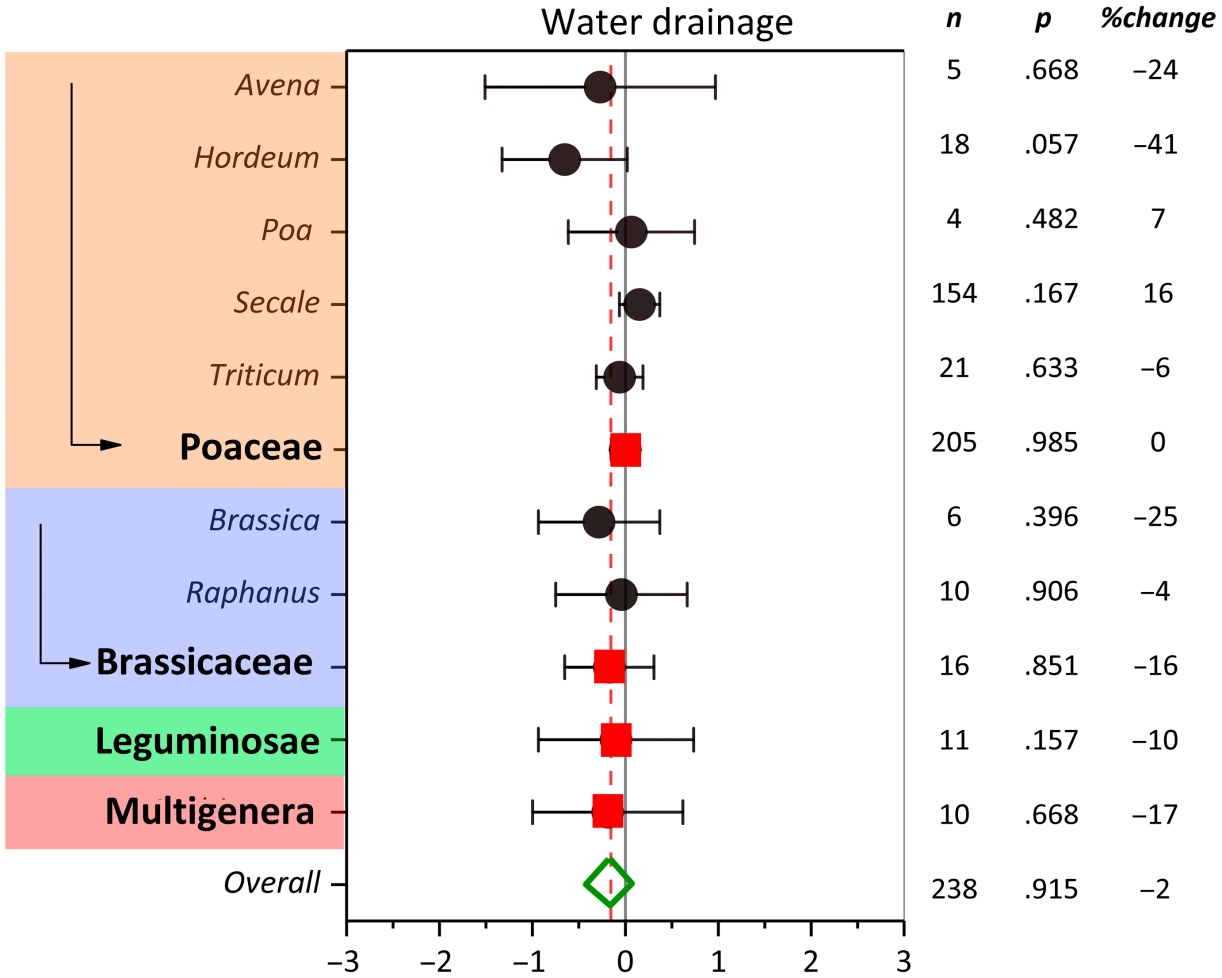
Weighted summary effect sizes (ln(*R*)) and 95% confidence intervals for cover crop effect on water drainage as influenced by cover crop family and suborder plant genus. %Change refers to the effect size differences from the overall mean. *n* is the number of studies. *p*‐value <.05 indicates that the summary effect size is different than zero.

**FIGURE 6:**
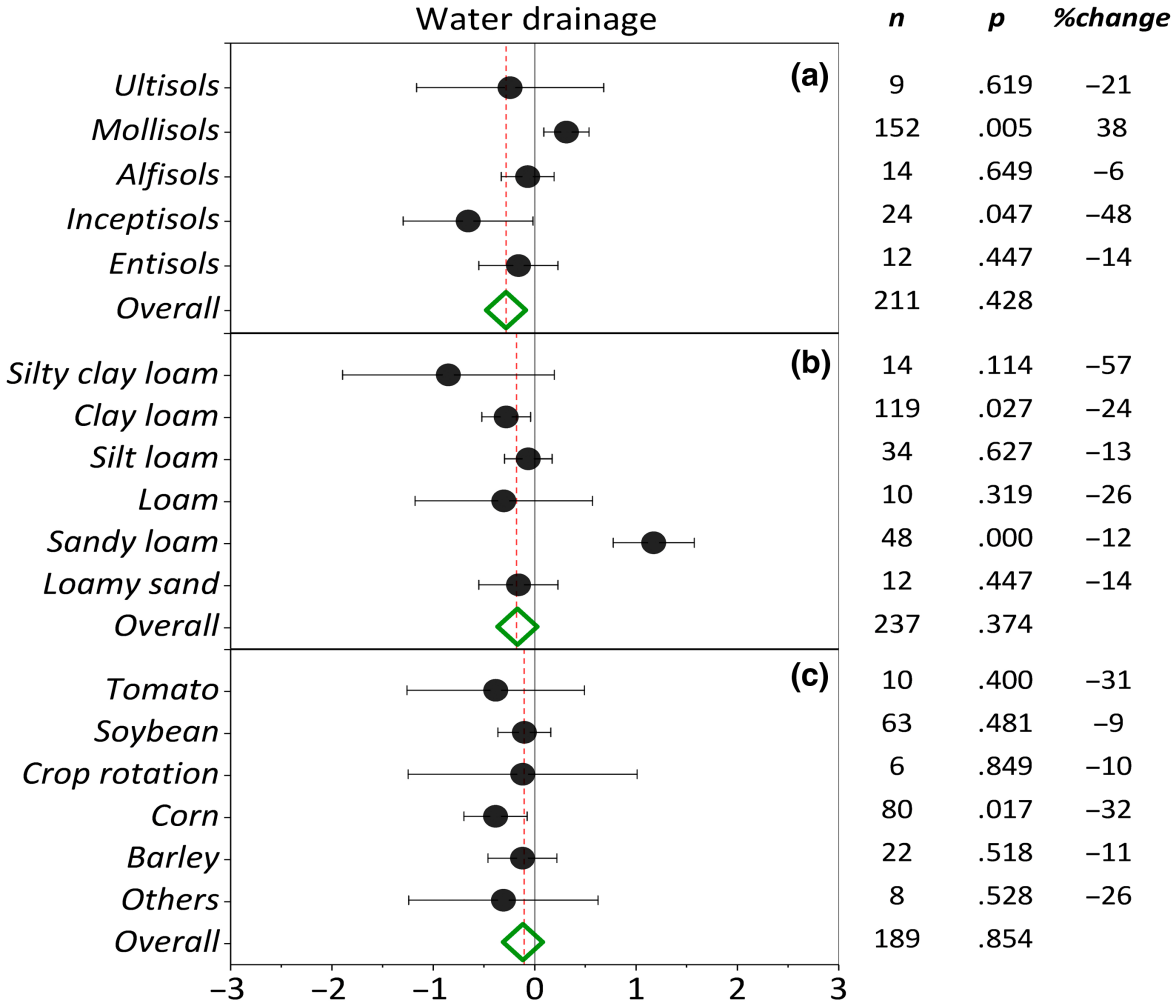
Weighted summary effect sizes (ln(*R*)) and 95% confidence intervals for cover crop effect on water drainage as influenced by (a) soil order, (b) soil texture, and (c) main crop. %Change refers to the effect size differences from the overall mean. *n* is the number of studies. *p*‐value <.05 indicates that the summary effect size is different than zero.

**FIGURE 7:**
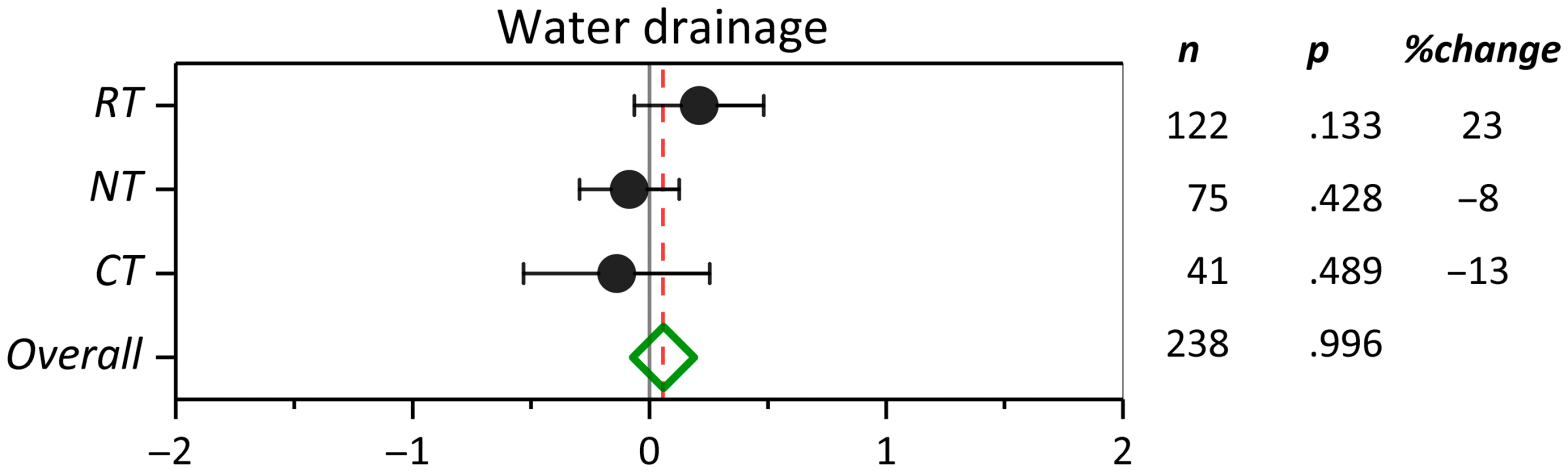
Weighted summary effect sizes (ln(*R*)) and 95% confidence intervals for cover crop effect on water drainage as influenced by tillage practices. %Change refers to the effect size differences from the overall mean. *n* is the number of studies. *p*‐value <.05 is the significance metric at 95% confidence interval.

The overall summary effect (ES = 0.068, CI = 0.004) of cover crops on water drainage was not significant. Cover cropping reduced water drainage only by 2% compared with NCC. Moderator analysis indicated that the effect size of water drainage was not significant among cover crop families (Figure 4). Among cover crop families and genera, *Hordeum* was the only genus that significantly reduced water drainage (*p* = .057) (Figure 5). *Hordeum, Brassica, and Avena* cover crop genera with 41, 25, and 24% change exhibited the greatest reduction in water drainage by cover cropping. An increase in observation counts resulted in narrower confidence intervals but did not affect the response ratios. Further analyses of moderator variables indicated no significant evidence of soil order, soil texture, and main crop controls on water drainage by cover cropping (Figure 6). Among soil orders, cover cropping only on Inceptisols resulted in a significant (*p* = .047) decrease in water drainage, while no trend existed in water drainage from soils at climax evolution (Ultisols) to newly formed (Entisols) soils (Figure 6a). In contrast to other soil orders, cover cropping on Mollisols resulted in a significant increase in water drainage. Cover crops tended to reduce water drainage moving from sandy to clayey soils (Figure 6b), although the effect size was significant only for clay loam (*p* = .027) and sandy loam (*p* = .000) soil textural classes. Among main crops, corn cropping systems with 32% (*p* = .017) reduction in water drainage showed the greatest CC/NCC (cover crop/no cover crop) effect size (EF = .441). Based on the available data, soil tillage did not significantly influence the effectiveness of cover crops in modifying water drainage (Figure 7). However, the ES pattern among tillage systems in water drainage reconciled well with the ES pattern among tillage systems in nitrate leaching. Cover cropping in CT system resulted in a −13% change in water drainage compared with −8% and 23% changes inflicted by NT and RT systems, respectively.

### 3.4 Meta‐regression analysis

**FIGURE 8:**
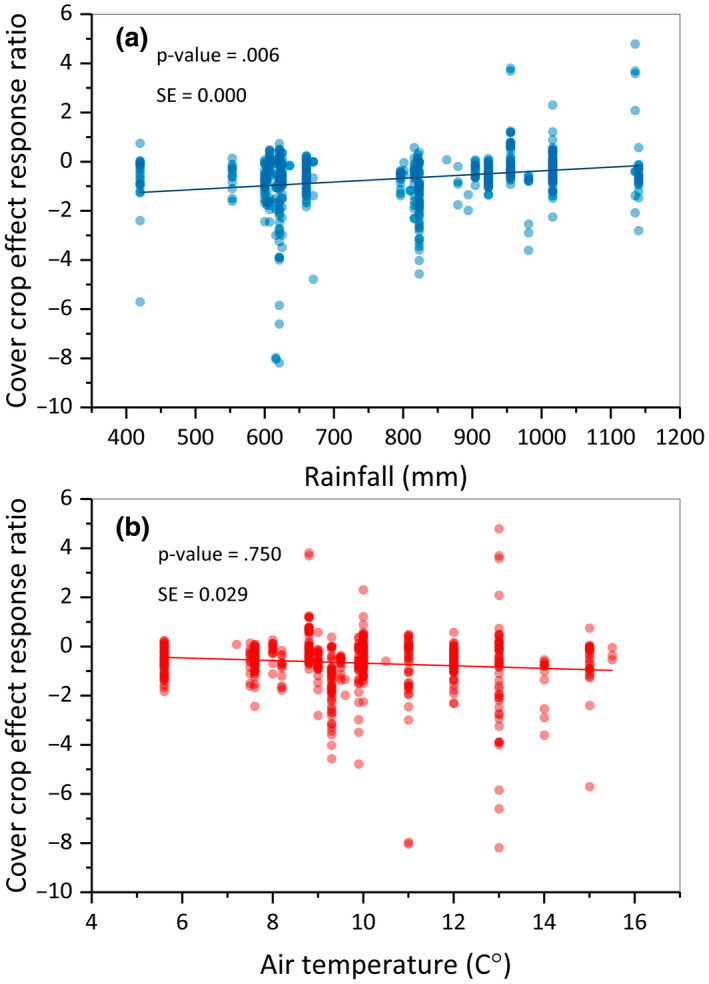
Natural log of the response ratio of CC/NCC as one unit increase in (a) rainfall amount and (b) air temperature.

In addition to the moderator analysis for the categorical variables, we assessed the associations between effect sizes and two numerical moderators—mean annual rainfall and air temperature (Figure 8). The model statistics have been provided in Table 3. The *p*‐values associated with *Q*‐statistics (*p* = .006) indicate that rainfall affects the effect size of nitrate leaching. The magnitude of this effect can be obtained by subtracting the product of ln(*R*) by absolute rainfall amount from the intercept. This relationship (regression slope) indicates that the ln(*R*) increases by 0.001 per unit increase in rainfall amount, meaning that the efficacy of cover crops in reducing nitrate leaching is reduced by 0.001 per unit increase in rainfall. The corresponding meta‐regression analysis for air temperature indicates no significant effect (*p* = .750) on the effectiveness of cover crop in reducing nitrate leaching.

**TABLE 3:** Regression moderators examined for the summary effects showing significant true heterogeneity among effect sizes

	Effect size and 95% interval					Test for the model		
	ln(*R*)^a^	SE	LL	UL	*p*‐value	*Q*	df	*p*‐value
Slope (rainfall)	0.001	0.000	0.000	0.002	.006	7.580	1.000	.006
Intercept	−1.556	0.300	−2.144	−0.968	.000			
Slope (temp)	−0.009	0.029	−0.065	0.047	.754	0.100	1.000	.750
Intercept	−0.665	0.278	−1.210	−0.119	.017			

Abbreviation: SE, standard error.

^a^ Ln(*R*) is the log effect size.

## 4 DISCUSSION

### 4.1 Overall summary effects

Our meta‐analysis showed that cover cropping reduce nitrate leaching from agricultural systems. The overall magnitude of this reduction across Poaceae, Brassicaceae, Leguminosae, and Asteraceae families and multigenera cover crops was 68%, compared with winter fallow (NCC). Previous studies in the literature have assessed the impact of cover crops on nitrate leaching between two broad categories of Leguminosae and non‐Leguminosae cover crops. Tonitto et al. (2006) reported a 70% decrease in nitrate leaching by non‐Leguminosae cover crops and 40% reduction due to planting Leguminosae species. Quemada et al. (2013) determined that non‐Leguminosae cover crops reduce nitrate leaching by 50% while non‐Leguminosae species did not show any effect. In the meta‐analysis conducted by Thapa et al. (2018) on 22 studies, replacing winter fallow with non‐Leguminosae cover crops reduces nitrate reduced leaching by 56% compared with winter fallow (NCC) while Leguminosae and legume–nonlegume mixtures did not significantly affect the nitrate leaching. The mechanism through which cover crops may affect nitrate leaching can be summarized by three main processes: (i) increasing solute conductivity through modifying the soil structure, especially by increasing number of preferential pathways due to increased root biomass; (ii) active uptake of nitrate during the vegetative growth stage (early fall); and (iii) microbial immobilization due to increased biomass input by cover crops (Li, Sørensen, et al., 2020; Quemada et al., 2013; Thapa et al., 2018). Nitrate is relatively unreactive, thus transfers in the soil profile at an almost equivalent rate to water flux. Water percolation is the leading mechanism in nitrate leaching, thus practices that influence water drainage inevitably affect nitrate leaching as well. Most studies in the literature that used lysimeters or suction cups to measure nitrate leaching, also reported the amount of drainage water. We took this opportunity to evaluate whether modifying water drainage is among the mechanisms through which cover crops reduce nitrate leaching. Thus, we assessed the CC/NCC of water drainage as the second effect size across identical moderators. Cover crops did not significantly change water drainage compared with NCC. This result indicates that soil structural modifications, especially in the short‐term and the consequent reductions in water and solute transport may not be a primary mechanism in nitrate leaching control by cover crops. The short experimental durations in almost all included publications (<5‐year) are likely a reason for the lack of significant differences in water drainage between NCC and CC practices since soil structural modification is a time‐demanding process (Nouri et al., 2020).

### 4.2 Cover crop families and genera

The meta‐analysis indicates that with the exception for Asteraceae (*p* > .05), cover crops from all other plant families (i.e., Brassicaceae, Poaceae, Leguminosae, and Multigenera) effectively reduce nitrate leaching, even though discrepancies existed among subfamily plant genera. Cover crops genera under Brassicaceae and Poaceae families exhibited greater overall summary effect size than Leguminosae, Asteraceae, and Multigenera cover crops. This result aligns with the results from the previous studies (Quemada et al., 2013; Thapa et al., 2018) which reported a greater potential for non‐Leguminosae (without further differentiation) than Leguminosae cover crops in reducing nitrate leaching. In contrast, in a critical review, Abdalla et al. (2019) reported no significant difference between Leguminosae, non‐Leguminosae, and mixed species cover crops on reducing nitrate leaching. Due to a limited body of literature, the effectiveness of cover crops at reducing nitrate leaching has been generally investigated within two broad categories of Leguminosae and non‐Leguminosae plant species. Conceivably, this has led to the shortage of information regarding the true potential of other large cover crop families such as Brassicaceae, Poaceae, and Asteraceae in nitrate removal from agroecosystems. The current study's most recent body of literature reveals that the Leguminosae cover crops similarly reduce nitrate leaching, although to a lower extent than non‐Leguminosae cover crops. The ability of the legumes to fix atmospheric nitrogen makes the plant a source rather than a sink for nitrogen, thereby lowering the potential of Leguminosae cover crop species in nitrate removal (Greenwood et al., 1990). Multigenera cover crops were as effective as Poaceae monoculture, but less effective than Brassicaceae in reducing nitrate leaching (White et al., 2017). The high potential of Brassicaceae cover crops can be attributed to the plant's high nitrogen demand during the vegetative growth stage (Xie & Kristensen, 2016). The deep rooting system of Brassicaceae cover crops maybe another reason for a greater potential of nitrogen removal (Kemper et al., 2020). Relatively greater C:N ratio in Brassicaceae than Leguminosae species leads to a longer N retention time, thereby reducing residual soil nitrate that is prone to leaching during the fallow period (Génard et al., 2016).

### 4.3 Soil order

Cover crop effect on the reduction of nitrate leaching across soil orders followed the order: Histosols > Ultisols > Inceptisols > Entisols > Mollisols > Alfisols. Among soil orders, cover cropping in Ultisols, Histosols, and Inceptisols exhibited greater influence on reducing nitrate leaching. In spite of distinct morphological features, all these soil orders are typical of humid and subhumid climatic regions where precipitation generally exceeds evaporation. The surface horizon in Ultisols is generally well‐drained and coarse‐textured surface solum typically has insufficient clay particles or organic matter as an organic source of nitrogen or adsorbent sites. Lessivage makes the coarse‐textured topsoil highly prone to nitrate leaching by mass flow. It should be noted that greater than currently available nine studies are required for more solid inferences about the role of cover crops on nitrate leaching in Ultisols. Inceptisols, despite low pedogenic development are not typical of aridic moisture regime (Baillie, 2001). They may even occasionally develop placic horizon resulting from oxygen exudation by plant roots under saturated conditions. Similarly, Histosols, are highly conductive lowland organic soils that are frequently anaerobic due to high water tables or low evaporation. The cool weather reduces the activity of denitrifying bacteria in Histosols and reduces nitrogen immobilization. Additionally, surface charges of soil colloids are pH dependent (Lazaratou et al., 2020). The adsorption of ammonium (NH_4_^+^) cations by adsorbent soil surfaces is crucial for reducing nitrification and mobility of nitrate anions (Li, Zeng, et al., 2020). The adsorption of ammonium decreases with increases in soil acidity due to increasing rainfall, which tends to leach bases out of the soil profile (Brady et al., 2008). It has been shown that the efficiency of ammonium removal from the soil solution increases by 20%–30% when soil pH increases from 2 to 7 (Alshameri et al., 2018). Therefore, insufficient N retention in immobile forms, water conductive topsoil, intense terrestrial water cycle (Huntington, 2010), and proximity to water table likely make Ultisols, Histosols, and Inceptisols more prone to nitrate leaching. This is a possible reason for high efficacy of cover crops reducing nitrate leaching compared with other soil orders. The CC/NCC effect size results based on soil order moderator in Alfisols were uncertain (Figure 3). The large confidence interval and nonsignificant effect is likely due to the limited number of observations associated with this soil order.

### 4.4 Soil texture

Overall, the increase in the effectiveness of cover crops in reducing nitrate leaching with decreasing clay content is evident. This trend aligns with soil water conductivity order among six soil textural classes, a major driving force for nitrate leaching. This result indicates that cover crop efficacy in excess nitrate uptake is higher when soil texture is more prone to nitrate leaching. Our findings are in agreement with the previous meta‐analysis that reported a decreasing trend of cover crops efficacy in reducing nitrate leaching across coarse‐, medium‐, and fine‐textured soils (Thapa et al., 2018). In contrast, in a critical review, Abdalla et al. (2019) found no significant soil texture effect on cover crops' efficacy in reducing nitrate leaching.

### 4.5 Main cropping system

Horticultural crops—that is, tomato, potato, and sugar beet showed above‐average responses in the reduction of nitrate leaching by planting cover crops. Those crops are generally grown on well‐drained medium‐ to coarse‐textured soils (Tei et al., 2017). Thus, the reason for better outcome can be partially relevant to the greater efficacy of cover crops to reduce nitrate leaching under coarse‐textured soils as discussed in the subsection 4.4. Another reason could be the fact that compared with most of the field crops, vegetable crops produce lower above‐ground biomass and less extensive root systems, reducing the area explored for nitrogen uptake (Greenwood et al., 1982). Thus, higher residual nitrogen may be present in the field after harvest, particularly at greater soil depths. Subsequent cover crops with more extensive root systems are likely to take up the excess N. The interaction between main crop and cover crop in terms of common nutrient pool, biomass quality, and allelochemicals may also affect cover crop performance in reducing nitrate leaching (Génard et al., 2016).

### 4.6 Tillage management

Conventional tillage demonstrated a greater potential for reducing nitrate leaching by cover crops compared with NT and RT practices. This result is in contrast with Thapa et al. (2018) who reported an almost 25% greater potential of RT than CT in reducing nitrate leaching by cover crops. However, consistent with our results, Daryanto et al. (2017) reported CT with greater potential to reduce nitrate leaching under cover crops. Nonetheless, NT management was not compared to other tillage systems in either of the previous analyses. The possible reason for the greater CC/NCC effect size under CT than NT is the difference of flow pathways in two tillage systems. Specifically, CT creates a more homogenous distribution of soil pores within the tilled zone which encourages the matrix flow in the soil profile. However, NT especially when managed for longer periods tend to develop sporadic biopores (interconnected macropores) created by rooting system or the activity of soil fauna (Nouri et al., 2019). However, it may be of concern mostly in medium‐ to heavy‐textured soils where fine soil particles permit the soil structural evolution under the lack of tillage disturbance.

### 4.7 Meta‐regression

Meta‐regression analysis indicates that nitrate leaching is reduced more by cover crops when planted in dry than wet climates. Rainfall and temperature gradients influence the effectiveness of cover crops in reducing nitrate leaching by altering soil water availability as well as the soil evolution and management strategies. Our analysis indicates that the cumulative effect of climate‐induced changes in immediate water availability, soil pedology, soil texture, management decision, and main crop diversity is responsible for greater cover crop effectiveness. Likewise, air temperature is an essential climatic factor determining both N cycling and cover crop establishment. Higher off‐season temperature allows the timely establishment and healthy standing of cover crops and microbial root colonization which is an important auxiliary mechanism aiding cover crops to reduce nitrate leaching by N transformation and provision to cover crops.

### 4.8 Cover crop effect on water drainage

The results indicated no significant effect of cover crops in altering water drainage. This outcome indicates that the reduction in nitrate leaching under cover crops as demonstrated in previous sections is likely not driven by the reduction in water percolation. Also, cover crops did not increase water drainage. There are contradictory results in the literature regarding the effect of cover crops on water drainage. Some studies have reported an increase in water drainage due to cover cropping (Alletto et al., 2012; Meyer et al., 2020; Rakotovololona et al., 2019)—others have reported a reduction (Meisinger et al., 1991; Meyer et al., 2020), while some observed no effect (Qi et al., 2011; Ward et al., 2012). In conclusion, due to the limited number of experimental data, the lack of significance between cover cropping and water drainage in the current study may not necessarily indicate the lack of true relationship. Further studies are needed to support this result or reveal another relationship.

## 5 CONCLUSION

This meta‐analysis revealed a significant impact of cover crops on the reduction of nitrate leaching. The effectiveness of cover crops in reducing nitrate leaching varied largely across cover crop families and genera, main crops, soils, agronomic management, and climates. The overall conditions that generate high nitrate leaching from agroecosystems, appeared to also increase the cover crop efficacy at reducing nitrate leaching. For instance, the greatest cover crop efficacy was observed in coarse‐textured soils, vegetable cropping systems, and tilled soils. The nonsignificant effect of cover crops on water drainage suggests that nitrate leaching control by cover crops is unlikely exerted through reducing water drainage. The current meta‐analysis provides insight into the climatic and terrestrial factors as well as management practices that influence the cover crop effect on nitrate leaching. It should be acknowledged that there is a considerable shortage of research articles in the literature regarding the direct measurement of nitrogen leaching under cover cropping scenarios. Although a clear trend of reduction in nitrate leaching by cover cropping is evident across crop species and soils, further studies can more extensively and reliably guide farmers', stakeholders', and policymakers' decisions toward the best cover crop management practices. For example, the effect of the number of cover cropping years, planting date, soil biology, etc. is yet to be fully understood. Also, future studies are expected to help better understand the effect of changing climatic patterns (including long‐term trends and extreme events) on cover crop efficacy in reducing nitrate leaching.

## CONFLICT OF INTEREST

The authors declare no conflict of interest.

## Supporting information


Appendix S1

## Data Availability

The datasets relevant to the current study are available at https://zenodo.org/record/6547522#.Yn7BoujMJPY.
