# Supplementary material for: When do cover crops reduce nitrate leaching? A global meta‐analysis
Source: Glob Chang Biol. 2022 Jun 8;28(15):4736–49. doi: 10.1111/gcb.16269 (PMC9328130; doi:10.1111/gcb.16269)
Supplement: Supplementary file 1 — Appendix S1 [file GCB-28-4736-s001.docx]

**Appendix**

**When Do Cover Crops Reduce Nitrate Leaching? A Global Meta-Analysis**

Amin Nouri ^a^, Scott Lukas ^a*^, Shikha Singh ^a^, Surendra Singh ^b^, Stephen Machado ^b^

^a^Hermiston Agricultural Research and Extension Center, Oregon State University, 2121 S 1^st^ street, Hermiston, OR 97838, USA.

^b^Columbia Basin Agricultural Research Center, Oregon State University, 48037 Tubbs Ranch Rd., Adams, OR 97810, USA.

**The list of meta-analysis articles**

Beckwith, C., Cooper, J., Smith, K., and Shepherd, M. (1998). Nitrate leaching loss following application of organic manures to sandy soils in arable cropping. I. Effects of application time, manure type, overwinter crop cover and nitrification inhibition. Soil Use and Management 14, 123-130.

Brandi‐Dohrn, F. M., Hess, M., Selker, J. S., Dick, R. P., Kauffman, S. M., and Hemphill Jr, D. D. (1997). "Nitrate leaching under a cereal rye cover crop," Rep. No. 0047-2425. Wiley Online Library.

Campiglia, E., Mancinelli, R., Radicetti, E., and Marinari, S. (2011). Legume cover crops and mulches: effects on nitrate leaching and nitrogen input in a pepper crop (Capsicum annuum L.). Nutrient Cycling in Agroecosystems 89, 399-412.

Catt, J., Howse, K., Christian, D., Lane, P., Harris, G., and Goss, M. (1998). Strategies to decrease nitrate leaching in the Brimstone Farm Experiment, Oxfordshire, UK, 1988–1993: the effects of winter cover crops and unfertilised grass leys. Plant and soil 203, 57-69.

Constantin, J., Mary, B., Laurent, F., Aubrion, G., Fontaine, A., Kerveillant, P., and Beaudoin, N. (2010). Effects of catch crops, no till and reduced nitrogen fertilization on nitrogen leaching and balance in three long-term experiments. Agriculture, ecosystems & environment 135, 268-278.

Daigh, A. L., Zhou, X., Helmers, M. J., Pederson, C. H., Horton, R., Jarchow, M., and Liebman, M. (2015). Subsurface drainage nitrate and total reactive phosphorus losses in bioenergy‐based prairies and corn systems. Journal of Environmental Quality 44, 1638-1646.

De Notaris, C., Rasmussen, J., Sørensen, P., and Olesen, J. E. (2018). Nitrogen leaching: A crop rotation perspective on the effect of N surplus, field management and use of catch crops. Agriculture, Ecosystems & Environment 255, 1-11.

Dougherty, B. W., Pederson, C. H., Mallarino, A. P., Andersen, D. S., Soupir, M. L., Kanwar, R. S., and Helmers, M. J. (2019). Midwestern cropping system effects on drainage water quality and crop yields. Journal of Environmental Quality.

Drury, C., Tan, C., Welacky, T., Reynolds, W., Zhang, T., Oloya, T., McLaughlin, N., and Gaynor, J. (2014). Reducing nitrate loss in tile drainage water with cover crops and water‐table management systems. Journal of Environmental Quality 43, 587-598.

Farneselli, M., Benincasa, P., Tosti, G., Guiducci, M., and Tei, F. (2020). Combining Green Manuring and Fertigation Maximizes Tomato Crop Yield and Minimizes Nitrogen Losses. Agronomy 10, 977.

Feaga, J. B., Selker, J. S., Dick, R. P., and Hemphill, D. D. (2010). Long‐term nitrate leaching under vegetable production with cover crops in the Pacific Northwest. Soil Science Society of America Journal 74, 186-195.

Fraser, P., Curtin, D., Harrison-Kirk, T., Meenken, E., Beare, M., Tabley, F., Gillespie, R., and Francis, G. (2013). Winter nitrate leaching under different tillage and winter cover crop management practices. Soil Science Society of America Journal 77, 1391-1401.

Gabriel, J., Muñoz-Carpena, R., and Quemada, M. (2012). The role of cover crops in irrigated systems: Water balance, nitrate leaching and soil mineral nitrogen accumulation. Agriculture, Ecosystems & Environment 155, 50-61.

Griffith, K. E., Young, E. O., Klaiber, L. B., and Kramer, S. R. (2020). Winter rye cover crop impacts on runoff water quality in a northern New York (USA) tile-drained maize agroecosystem. Water, Air, & Soil Pollution 231, 1-16.

Heinrich, A., Smith, R., and Cahn, M. (2014). Winter-killed cereal rye cover crop influence on nitrate leaching in intensive vegetable production systems. HortTechnology 24, 502-511.

Herrera, J. M., Feil, B., Stamp, P., and Liedgens, M. (2010). Root growth and nitrate‐nitrogen leaching of catch crops following spring wheat. Journal of environmental quality 39, 845-854.

Herrera, J. M., and Liedgens, M. (2009). Leaching and utilization of nitrogen during a spring wheat catch crop succession. Journal of environmental quality 38, 1410-1419.

Hooker, K., Coxon, C., Hackett, R., Kirwan, L., O'Keeffe, E., and Richards, K. (2008). Evaluation of cover crop and reduced cultivation for reducing nitrate leaching in Ireland. Journal of environmental Quality 37, 138-145.

Kaspar, T., Jaynes, D., Parkin, T., and Moorman, T. (2007). Rye cover crop and gamagrass strip effects on NO3 concentration and load in tile drainage. Journal of environmental quality 36, 1503-1511.

Kaspar, T., Jaynes, D., Parkin, T., Moorman, T., and Singer, J. (2012). Effectiveness of oat and rye cover crops in reducing nitrate losses in drainage water. Agricultural Water Management 110, 25-33.

Macdonald, A., Poulton, P., Howe, M., Goulding, K., and Powlson, D. (2005). The use of cover crops in cereal-based cropping systems to control nitrate leaching in SE England. Plant and Soil 273, 355-373.

Martinez, J., and Guiraud, G. (1990). Alysimeter study of the effects of a ryegrass catch crop, during a winter wheat/maize rotation, on nitrate leaching and on the following crop. Journal of Soil Science 41, 5-16.

Meisinger, J. J., and Ricigliano, K. A. (2017). Nitrate leaching from winter cereal cover crops using undisturbed soil‐column lysimeters. Journal of Environmental Quality 46, 576-584.

Norberg, L., and Aronsson, H. (2020). Effects of cover crops sown in autumn on N and P leaching. Soil Use and Management 36, 200-211.

Pedersen, B. N., Eriksen, J., Christensen, B. T., and Sørensen, P. (2021). Fertilizer replacement value and leaching of nitrogen applied to spring barley in cattle deep litter: a 3-year lysimeter study. Soil and Tillage Research 209, 104954.

Premrov, A., Coxon, C. E., Hackett, R., Kirwan, L., and Richards, K. G. (2014). Effects of over-winter green cover on soil solution nitrate concentrations beneath tillage land. Science of the Total Environment 470, 967-974.

Qi, Z., Helmers, M. J., Christianson, R. D., and Pederson, C. H. (2011). Nitrate‐nitrogen losses through subsurface drainage under various agricultural land covers. Journal of environmental quality 40, 1578-1585.

Rasse, D. P., Ritchie, J. T., Peterson, W. R., Wei, J., and Smucker, A. J. (2000). "Rye cover crop and nitrogen fertilization effects on nitrate leaching in inbred maize fields," Rep. No. 0047-2425. Wiley Online Library.

Ritter, W., Scarborough, R. W., and Chirnside, A. (1998). Winter cover crops as a best management practice for reducing nitrogen leaching. Journal of Contaminant Hydrology 34, 1-15.

Shepherd, M. (1999). The effectiveness of cover crops during eight years of a UK sandland rotation. Soil Use and Management 15, 41-48.

Shepherd, M., and Webb, J. (1999). Effects of overwinter cover on nitrate loss and drainage from a sandy soil: consequences for water management? Soil use and management 15, 109-116.

Singh, G., Williard, K. W. J., and Schoonover, J. E. (2018). Cover Crops and Tillage Influence on Nitrogen Dynamics in Plant-Soil-Water Pools. Soil Science Society of America Journal 82, 1572-1582.

Strock, J. S., Porter, P. M., and Russelle, M. (2004). Cover cropping to reduce nitrate loss through subsurface drainage in the northern US Corn Belt. Journal of environmental quality 33, 1010-1016.

Tosti, G., Benincasa, P., Farneselli, M., Tei, F., and Guiducci, M. (2014). Barley–hairy vetch mixture as cover crop for green manuring and the mitigation of N leaching risk. European Journal of Agronomy 54, 34-39.

Vogeler, I., Hansen, E. M., Thomsen, I. K., and Ostergaard, H. S. (2019). Legumes in catch crop mixtures: Effects on nitrogen retention and availability, and leaching losses. Journal of Environmental Management 239, 324-332.

Walmsley, D. C., Siemens, J., Kindler, R., Kaiser, K., Saunders, M., Fichtner, A., Kaupenjohann, M., and Osborne, B. A. (2018). Reduced nitrate leaching from an Irish cropland soil under non-inversion tillage with cover cropping greatly outweighs increased dissolved organic nitrogen leaching. Agriculture Ecosystems & Environment 265, 340-349.

Waring, E. R., Lagzdins, A., Pederson, C., and Helmers, M. J. (2020). Influence of no-till and a winter rye cover crop on nitrate losses from tile-drained row-crop agriculture in Iowa. Journal of Environmental Quality 49, 292-303.

Wen, Y., Zang, H. D., Ma, Q. X., Freeman, B., Chadwick, D. R., Evans, C. D., and Jones, D. L. (2020). Impact of water table levels and winter cover crops on greenhouse gas emissions from cultivated peat soils. Science of the Total Environment 719.

Weyers, S., Thom, M., Forcella, F., Eberle, C., Matthees, H., Gesch, R., Ott, M., Feyereisen, G., Strock, J., and Wyse, D. (2019). Reduced Potential for Nitrogen Loss in Cover Crop-Soybean Relay Systems in a Cold Climate. Journal of Environmental Quality 48, 660-669.

Yang, W., Feng, G., Adeli, A., Kersebaum, K., Jenkins, J. N., and Li, P. F. (2019). Long-term effect of cover crop on rainwater balance components and use efficiency in the no-tilled and rainfed corn and soybean rotation system. Agricultural Water Management 219, 27-39.

Zhao, J., De Notaris, C., and Olesen, J. E. (2020). Autumn-based vegetation indices for estimating nitrate leaching during autumn and winter in arable cropping systems. Agriculture Ecosystems & Environment 290.
